# *catena*-Poly[[aqua­bis­{4-[2-(2,4-dioxopentan-3-yl­idene)hydrazin-1-yl]benzo­ato-κ*O*}copper(II)]-μ-*N*,*N*-diethyl­pyridine-3-carboxamide-κ^2^ *N* ^1^:*O*]

**DOI:** 10.1107/S1600536812000025

**Published:** 2012-01-11

**Authors:** Abel M. Maharramov, Vusala I. Mardanova, Famil M. Chyraqov, Atash V. Gurbanov, Seik Weng Ng

**Affiliations:** a Department of Organic Chemistry, Baku State University, Baku, Azerbaijan; b Department of Chemistry, University of Malaya, 50603 Kuala Lumpur, Malaysia, and, Chemistry Department, Faculty of Science, King Abdulaziz University, PO Box 80203 Jeddah, Saudi Arabia

## Abstract

The Cu^II^ atom in the title compound, [Cu(C_12_H_11_N_2_O_4_)_2_(C_10_H_14_N_2_O)(H_2_O)]_*n*_, lies in a square plane defined by the O atoms of the carboxyl­ate ions, the N atom of the *N*-heterocycle and the water mol­ecule. Coordination by an amido O atom of an adjacent *N*-heterocycle in the apical direction leads to a polymeric chain running along [01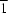]. The chain motif is consolidated by hydrogen bonds involving the water mol­ecule; the water mol­ecule is a hydrogen-bond donor to the free carbonyl atoms of the carboxyl­ate ions. Intra­molecular N—H⋯O hydrogen bonds also occur.

## Related literature

For a related structure, see: Maharramov *et al.* (2011 ▶).
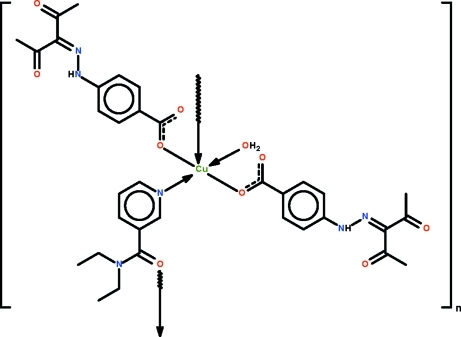


## Experimental

### 

#### Crystal data

[Cu(C_12_H_11_N_2_O_4_)_2_(C_10_H_14_N_2_O)(H_2_O)]
*M* *_r_* = 754.24
Monoclinic, 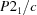

*a* = 17.0586 (8) Å
*b* = 8.5289 (4) Å
*c* = 24.8249 (12) Å
β = 101.431 (1)°
*V* = 3540.2 (3) Å^3^
*Z* = 4
Mo *K*α radiation
μ = 0.68 mm^−1^
*T* = 296 K
0.30 × 0.20 × 0.20 mm

#### Data collection

Bruker SMART APEX diffractometer
Absorption correction: multi-scan (*SADABS*; Sheldrick, 1996 ▶) *T*_min_ = 0.822, *T*_max_ = 0.876
40068 measured reflections
8845 independent reflections
6058 reflections with *I* > 2σ(*I*)
*R*_int_ = 0.052

#### Refinement

*R*[*F* ^2^ > 2σ(*F* ^2^)] = 0.045
*wR*(*F* ^2^) = 0.131
*S* = 1.00
8845 reflections
480 parameters
4 restraints
H atoms treated by a mixture of independent and constrained refinement
Δρ_max_ = 1.14 e Å^−3^
Δρ_min_ = −0.39 e Å^−3^

### 

Data collection: *APEX2* (Bruker, 2005 ▶); cell refinement: *SAINT* (Bruker, 2005 ▶); data reduction: *SAINT*; program(s) used to solve structure: *SHELXS97* (Sheldrick, 2008 ▶); program(s) used to refine structure: *SHELXL97* (Sheldrick, 2008 ▶); molecular graphics: *X-SEED* (Barbour, 2001 ▶); software used to prepare material for publication: *publCIF* (Westrip, 2010 ▶).

## Supplementary Material

Crystal structure: contains datablock(s) global, I. DOI: 10.1107/S1600536812000025/xu5432sup1.cif

Structure factors: contains datablock(s) I. DOI: 10.1107/S1600536812000025/xu5432Isup2.hkl

Additional supplementary materials:  crystallographic information; 3D view; checkCIF report

## Figures and Tables

**Table 1:** Selected bond lengths (Å)

Cu1—O1	1.9409 (16)
Cu1—O1*W*	1.9706 (18)
Cu1—O5	1.9278 (17)
Cu1—O9^i^	2.4505 (17)
Cu1—N5	2.032 (2)

Symmetry code: (i) 
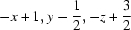
.

**Table 2:** Hydrogen-bond geometry (Å, °)

*D*—H⋯*A*	*D*—H	H⋯*A*	*D*⋯*A*	*D*—H⋯*A*
O1w—H11⋯O2^ii^	0.84 (1)	1.88 (1)	2.700 (2)	163 (4)
O1w—H12⋯O6^ii^	0.84 (1)	1.88 (1)	2.705 (3)	171 (3)
N1—H1⋯O3	0.88 (1)	1.84 (2)	2.552 (3)	138 (3)
N3—H3⋯O7	0.88 (1)	1.85 (2)	2.559 (3)	137 (3)

Symmetry code: (ii) 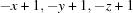.

**Fig. 1.**
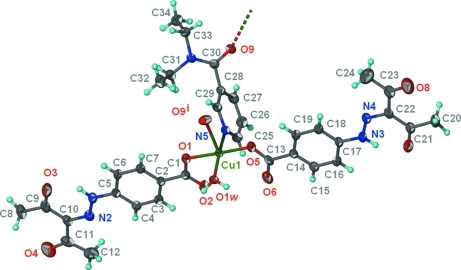
Thermal ellipsoid plot (Barbour, 2001) of the repeat unit of polymeric Cu(H2O)(C10H14N2O)(C12H11N2O4)2 at the 50% probability level; hydrogen atoms are drawn as spheres of arbitrary radius.

## supplementary crystallographic information

### Comment

We have been investigating the adducts of 3-diethylpridine-3-carboxamide with copper(II) salts; a previous study reported the copper bis(thienoylacetonate) adduct (Maharramov *et al.*, 2011). The reaction of the *N*-heterocycle with copper bis(4-[(2,4-dioxopentan-3-yl)diazenyl]benzoate in the presence of sodium bicarbonate yielded the title monoaqua mono-adduct (Scheme I). The Cu^II^ atom lies in a square plane defined by the O atom of the carboxylate ions, the N atom of the *N*-heterocycle and the water molecule; coordination by the amido O atom of an adjacent *N*-heterocycle leads to a linear chain running along [0 1 - 1]. The chain motif is consolidated by hydrogen bonds that involve the water molecule; the water molecule is hydrogen-bond donor to the the double-bond carbonyl O atom of the carboxylate ions (Table 1).

### Experimental

4-[(2,4-Dioxopentan-3-yl)diazenyl]benzoic acid and *N*,*N*-diethylpyridine-3-carboxamide (cardioamine) were purchased from a chemical supplier. The carboxylic acid (0.0248 g, 0.01 mol) in water (50 ml) and an excess of cardioamine (5 ml) were added to a solution of copper acetate hydrate (0.0156 g, 0.01 mol) in water (50 ml). Sodium bicarbonate (0.01 g) was also added. The green solution was filtered and then set aside for the growth of crystals within a week; yield 70%.

### Refinement

Carbon-bound H-atoms were placed in calculated positions [C–H 0.93 to 0.98 Å; *U*(H) 1.2 to 1.5*U*(C)] and were included in the refinement in the riding model approximation.

The water and amino- H-atoms were located in a difference Fourier map, and were refined with distance restraints of O–H 0.84±0.01 and N–H 0.88 + 0.01 Å; their temperature factors were refined.

Omitted were (1 0 0), (-2 1 0) and (4 1 9).

The final difference Fourier map had a peak at 2.27 Å from H25.

### Crystal data

**Table:**

[Cu(C_12_H_11_N_2_O_4_)_2_(C_10_H_14_N_2_O)(H_2_O)]	*F*(000) = 1572
*M**_r_* = 754.24	*D*_x_ = 1.415 Mg m^−^^3^
Monoclinic, *P*2_1_/*c*	Mo *K*α radiation, λ = 0.71073 Å
Hall symbol: -P 2ybc	Cell parameters from 5695 reflections
*a* = 17.0586 (8) Å	θ = 2.3–25.5°
*b* = 8.5289 (4) Å	µ = 0.68 mm^−^^1^
*c* = 24.8249 (12) Å	*T* = 296 K
β = 101.431 (1)°	Prism, green
*V* = 3540.2 (3) Å^3^	0.30 × 0.20 × 0.20 mm
*Z* = 4	

### Data collection

**Table:**

Bruker SMART APEX diffractometer	8845 independent reflections
Radiation source: fine-focus sealed tube	6058 reflections with *I* > 2σ(*I*)
graphite	*R*_int_ = 0.052
φ and ω scans	θ_max_ = 28.4°, θ_min_ = 1.9°
Absorption correction: multi-scan (*SADABS*; Sheldrick, 1996)	*h* = −22→22
*T*_min_ = 0.822, *T*_max_ = 0.876	*k* = −11→11
40068 measured reflections	*l* = −33→33

### Refinement

**Table:**

Refinement on *F*^2^	Primary atom site location: structure-invariant direct methods
Least-squares matrix: full	Secondary atom site location: difference Fourier map
*R*[*F*^2^ > 2σ(*F*^2^)] = 0.045	Hydrogen site location: inferred from neighbouring sites
*wR*(*F*^2^) = 0.131	H atoms treated by a mixture of independent and constrained refinement
*S* = 1.00	*w* = 1/[σ^2^(*F*_o_^2^) + (0.0692*P*)^2^ + 0.8919*P*] where *P* = (*F*_o_^2^ + 2*F*_c_^2^)/3
8845 reflections	(Δ/σ)_max_ = 0.001
480 parameters	Δρ_max_ = 1.14 e Å^−^^3^
4 restraints	Δρ_min_ = −0.39 e Å^−^^3^

### Fractional atomic coordinates and isotropic or equivalent isotropic displacement parameters (Å^2^)

**Table:**

	*x*	*y*	*z*	*U*_iso_*/*U*_eq_	
Cu1	0.493965 (16)	0.51736 (4)	0.599200 (11)	0.03106 (10)	
O1	0.38597 (10)	0.4339 (2)	0.58434 (7)	0.0385 (4)	
O2	0.36751 (10)	0.6083 (2)	0.51632 (8)	0.0442 (5)	
O3	−0.09251 (12)	0.1214 (3)	0.49904 (9)	0.0619 (6)	
O4	−0.21233 (15)	0.3194 (4)	0.35319 (11)	0.0928 (9)	
O5	0.60299 (10)	0.5906 (2)	0.61634 (7)	0.0422 (4)	
O6	0.58603 (12)	0.7412 (2)	0.54181 (8)	0.0535 (5)	
O7	1.06507 (12)	1.0617 (3)	0.62687 (9)	0.0588 (6)	
O8	1.18499 (14)	0.8684 (3)	0.77257 (11)	0.0837 (8)	
O9	0.47008 (12)	0.8486 (2)	0.81977 (7)	0.0452 (5)	
O1W	0.52061 (10)	0.3639 (2)	0.54628 (7)	0.0348 (4)	
H11	0.5605 (14)	0.383 (4)	0.5322 (14)	0.078 (12)*	
H12	0.4840 (13)	0.333 (4)	0.5210 (9)	0.057 (9)*	
N1	0.02254 (12)	0.2933 (3)	0.48185 (9)	0.0394 (5)	
H1	0.0014 (18)	0.230 (3)	0.5028 (11)	0.066 (11)*	
N2	−0.02628 (12)	0.3447 (3)	0.43803 (9)	0.0381 (5)	
N3	0.94860 (12)	0.8959 (3)	0.64613 (9)	0.0378 (5)	
H3	0.9672 (16)	0.968 (3)	0.6270 (10)	0.046 (8)*	
N4	0.99542 (13)	0.8552 (3)	0.69171 (9)	0.0387 (5)	
N5	0.45847 (12)	0.6968 (2)	0.64261 (8)	0.0327 (4)	
N6	0.38353 (12)	0.6457 (2)	0.80156 (8)	0.0354 (5)	
C1	0.34316 (14)	0.5016 (3)	0.54298 (10)	0.0348 (6)	
C2	0.25803 (14)	0.4482 (3)	0.52657 (10)	0.0321 (5)	
C3	0.20856 (15)	0.5110 (3)	0.48070 (10)	0.0361 (5)	
H3A	0.2287	0.5868	0.4602	0.043*	
C4	0.13017 (15)	0.4639 (3)	0.46470 (10)	0.0366 (6)	
H4	0.0973	0.5078	0.4340	0.044*	
C5	0.10120 (14)	0.3492 (3)	0.49539 (10)	0.0338 (5)	
C6	0.14959 (15)	0.2853 (3)	0.54144 (10)	0.0372 (6)	
H6	0.1295	0.2096	0.5620	0.045*	
C7	0.22789 (14)	0.3345 (3)	0.55682 (10)	0.0364 (6)	
H7	0.2606	0.2910	0.5876	0.044*	
C8	−0.22150 (17)	0.1321 (4)	0.44365 (15)	0.0579 (8)	
H8A	−0.2343	0.0675	0.4724	0.087*	
H8B	−0.2309	0.0743	0.4098	0.087*	
H8C	−0.2545	0.2241	0.4395	0.087*	
C9	−0.13527 (16)	0.1793 (3)	0.45812 (12)	0.0440 (6)	
C10	−0.10051 (15)	0.2932 (3)	0.42495 (11)	0.0386 (6)	
C11	−0.14498 (19)	0.3630 (4)	0.37323 (12)	0.0520 (8)	
C12	−0.1043 (2)	0.4850 (4)	0.34495 (14)	0.0651 (9)	
H12A	−0.1396	0.5178	0.3118	0.098*	
H12B	−0.0565	0.4418	0.3361	0.098*	
H12C	−0.0908	0.5736	0.3689	0.098*	
C13	0.62698 (14)	0.6884 (3)	0.58437 (11)	0.0356 (6)	
C14	0.71265 (14)	0.7415 (3)	0.60170 (10)	0.0328 (5)	
C15	0.74303 (15)	0.8525 (3)	0.57086 (11)	0.0377 (6)	
H15	0.7104	0.8939	0.5396	0.045*	
C16	0.82117 (15)	0.9025 (3)	0.58593 (11)	0.0399 (6)	
H16	0.8413	0.9769	0.5649	0.048*	
C17	0.86936 (14)	0.8412 (3)	0.63267 (10)	0.0342 (5)	
C18	0.83995 (15)	0.7296 (3)	0.66377 (11)	0.0418 (6)	
H18	0.8726	0.6882	0.6950	0.050*	
C19	0.76182 (15)	0.6802 (3)	0.64811 (11)	0.0397 (6)	
H19	0.7419	0.6049	0.6689	0.048*	
C20	1.18795 (18)	1.0784 (4)	0.68823 (14)	0.0606 (9)	
H20A	1.1998	1.1489	0.6608	0.091*	
H20B	1.2250	0.9925	0.6927	0.091*	
H20C	1.1924	1.1332	0.7225	0.091*	
C21	1.10464 (16)	1.0172 (3)	0.67061 (12)	0.0421 (6)	
C22	1.06958 (15)	0.9084 (3)	0.70478 (11)	0.0382 (6)	
C23	1.11461 (18)	0.8444 (4)	0.75726 (13)	0.0512 (7)	
C24	1.0711 (3)	0.7474 (6)	0.79232 (17)	0.0967 (15)	
H24A	1.1056	0.7282	0.8272	0.145*	
H24B	1.0557	0.6493	0.7744	0.145*	
H24C	1.0243	0.8026	0.7978	0.145*	
C25	0.44230 (16)	0.8393 (3)	0.62092 (11)	0.0418 (6)	
H25	0.4471	0.8563	0.5847	0.050*	
C26	0.41883 (17)	0.9619 (3)	0.65009 (12)	0.0440 (6)	
H26	0.4089	1.0603	0.6340	0.053*	
C27	0.41020 (15)	0.9366 (3)	0.70357 (11)	0.0394 (6)	
H27	0.3941	1.0176	0.7240	0.047*	
C28	0.42588 (14)	0.7884 (3)	0.72654 (10)	0.0323 (5)	
C29	0.44957 (14)	0.6726 (3)	0.69416 (10)	0.0336 (5)	
H29	0.4598	0.5729	0.7091	0.040*	
C30	0.42650 (14)	0.7627 (3)	0.78670 (10)	0.0320 (5)	
C31	0.32376 (16)	0.5548 (4)	0.76355 (12)	0.0465 (7)	
H31A	0.3136	0.6060	0.7280	0.056*	
H31B	0.2741	0.5545	0.7770	0.056*	
C32	0.3491 (2)	0.3856 (4)	0.75645 (15)	0.0634 (9)	
H32A	0.3077	0.3325	0.7312	0.095*	
H32B	0.3579	0.3332	0.7913	0.095*	
H32C	0.3976	0.3847	0.7423	0.095*	
C33	0.38618 (16)	0.6195 (3)	0.86053 (10)	0.0408 (6)	
H33A	0.4378	0.6529	0.8812	0.049*	
H33B	0.3807	0.5082	0.8670	0.049*	
C34	0.32118 (18)	0.7069 (4)	0.88098 (12)	0.0519 (7)	
H34A	0.3255	0.6871	0.9195	0.078*	
H34B	0.2699	0.6720	0.8614	0.078*	
H34C	0.3267	0.8172	0.8751	0.078*	

### Atomic displacement parameters (Å^2^)

**Table:**

	*U*^11^	*U*^22^	*U*^33^	*U*^12^	*U*^13^	*U*^23^
Cu1	0.02397 (14)	0.04335 (19)	0.02612 (15)	−0.00660 (12)	0.00558 (11)	−0.00480 (13)
O1	0.0271 (8)	0.0519 (11)	0.0363 (10)	−0.0081 (8)	0.0055 (7)	−0.0027 (8)
O2	0.0340 (9)	0.0530 (12)	0.0489 (11)	−0.0100 (8)	0.0161 (8)	0.0013 (9)
O3	0.0392 (11)	0.0739 (16)	0.0674 (14)	−0.0159 (10)	−0.0022 (10)	0.0218 (12)
O4	0.0618 (16)	0.122 (2)	0.0758 (17)	−0.0148 (16)	−0.0318 (14)	0.0199 (17)
O5	0.0276 (9)	0.0568 (12)	0.0419 (10)	−0.0116 (8)	0.0063 (8)	−0.0040 (9)
O6	0.0419 (11)	0.0602 (13)	0.0501 (12)	−0.0109 (10)	−0.0109 (9)	−0.0003 (10)
O7	0.0449 (11)	0.0732 (15)	0.0558 (13)	−0.0198 (11)	0.0035 (10)	0.0137 (11)
O8	0.0542 (15)	0.096 (2)	0.0856 (18)	−0.0173 (14)	−0.0224 (13)	0.0246 (15)
O9	0.0571 (12)	0.0449 (11)	0.0342 (10)	−0.0156 (9)	0.0103 (9)	−0.0121 (8)
O1W	0.0274 (9)	0.0501 (11)	0.0264 (9)	−0.0045 (8)	0.0041 (8)	−0.0046 (8)
N1	0.0294 (11)	0.0452 (13)	0.0409 (12)	−0.0081 (10)	0.0008 (10)	0.0043 (10)
N2	0.0331 (11)	0.0387 (12)	0.0413 (12)	−0.0009 (9)	0.0042 (9)	−0.0041 (9)
N3	0.0295 (11)	0.0415 (13)	0.0421 (12)	−0.0090 (9)	0.0061 (9)	−0.0005 (10)
N4	0.0328 (11)	0.0407 (12)	0.0417 (12)	−0.0065 (9)	0.0050 (10)	−0.0042 (10)
N5	0.0307 (10)	0.0380 (12)	0.0289 (10)	−0.0043 (9)	0.0044 (8)	−0.0034 (9)
N6	0.0334 (11)	0.0401 (12)	0.0308 (11)	−0.0040 (9)	0.0013 (9)	−0.0010 (9)
C1	0.0279 (11)	0.0422 (15)	0.0364 (13)	−0.0039 (10)	0.0116 (10)	−0.0107 (11)
C2	0.0269 (11)	0.0382 (13)	0.0324 (12)	−0.0055 (10)	0.0086 (10)	−0.0071 (10)
C3	0.0348 (12)	0.0394 (14)	0.0356 (13)	−0.0053 (11)	0.0105 (10)	−0.0015 (11)
C4	0.0331 (12)	0.0416 (14)	0.0332 (13)	−0.0031 (11)	0.0020 (10)	0.0007 (11)
C5	0.0275 (12)	0.0407 (14)	0.0333 (13)	−0.0057 (10)	0.0061 (10)	−0.0059 (10)
C6	0.0333 (13)	0.0419 (15)	0.0367 (14)	−0.0083 (11)	0.0079 (11)	0.0031 (11)
C7	0.0302 (12)	0.0440 (15)	0.0345 (13)	−0.0045 (11)	0.0049 (10)	0.0006 (11)
C8	0.0338 (15)	0.059 (2)	0.076 (2)	−0.0093 (13)	−0.0010 (15)	−0.0034 (16)
C9	0.0321 (13)	0.0421 (15)	0.0548 (17)	−0.0037 (11)	0.0018 (13)	−0.0067 (13)
C10	0.0317 (13)	0.0380 (14)	0.0420 (14)	−0.0002 (11)	−0.0024 (11)	−0.0051 (11)
C11	0.0528 (18)	0.0535 (18)	0.0440 (16)	0.0077 (14)	−0.0039 (14)	−0.0073 (14)
C12	0.083 (3)	0.059 (2)	0.0496 (18)	0.0086 (18)	0.0040 (17)	0.0061 (15)
C13	0.0285 (12)	0.0381 (14)	0.0392 (14)	−0.0038 (10)	0.0041 (11)	−0.0158 (11)
C14	0.0287 (12)	0.0382 (14)	0.0321 (12)	−0.0042 (10)	0.0076 (10)	−0.0104 (10)
C15	0.0344 (13)	0.0409 (14)	0.0361 (13)	−0.0021 (11)	0.0028 (11)	−0.0024 (11)
C16	0.0378 (14)	0.0411 (15)	0.0413 (15)	−0.0089 (11)	0.0094 (12)	0.0011 (12)
C17	0.0252 (11)	0.0391 (14)	0.0386 (13)	−0.0065 (10)	0.0069 (10)	−0.0071 (11)
C18	0.0322 (13)	0.0541 (17)	0.0371 (14)	−0.0097 (12)	0.0022 (11)	0.0045 (12)
C19	0.0324 (13)	0.0503 (16)	0.0363 (14)	−0.0105 (11)	0.0064 (11)	0.0015 (12)
C20	0.0432 (16)	0.076 (2)	0.061 (2)	−0.0259 (16)	0.0066 (15)	−0.0003 (17)
C21	0.0358 (13)	0.0438 (15)	0.0466 (16)	−0.0063 (12)	0.0078 (12)	−0.0058 (12)
C22	0.0324 (13)	0.0379 (14)	0.0425 (14)	−0.0046 (11)	0.0033 (11)	−0.0062 (11)
C23	0.0487 (17)	0.0465 (17)	0.0536 (18)	−0.0048 (13)	−0.0015 (14)	0.0024 (14)
C24	0.084 (3)	0.125 (4)	0.075 (3)	−0.022 (3)	0.000 (2)	0.047 (3)
C25	0.0422 (15)	0.0506 (17)	0.0319 (13)	−0.0044 (12)	0.0053 (11)	0.0031 (12)
C26	0.0452 (15)	0.0383 (15)	0.0464 (16)	0.0037 (12)	0.0042 (13)	0.0068 (12)
C27	0.0360 (13)	0.0376 (14)	0.0428 (15)	0.0029 (11)	0.0032 (11)	−0.0066 (11)
C28	0.0278 (12)	0.0359 (13)	0.0321 (12)	−0.0019 (10)	0.0031 (10)	−0.0036 (10)
C29	0.0332 (12)	0.0350 (13)	0.0315 (12)	−0.0013 (10)	0.0037 (10)	−0.0015 (10)
C30	0.0315 (12)	0.0323 (13)	0.0324 (12)	0.0030 (10)	0.0067 (10)	−0.0035 (10)
C31	0.0357 (14)	0.0605 (19)	0.0407 (15)	−0.0126 (13)	0.0016 (12)	−0.0046 (13)
C32	0.071 (2)	0.053 (2)	0.068 (2)	−0.0277 (17)	0.0191 (18)	−0.0169 (16)
C33	0.0461 (15)	0.0406 (15)	0.0345 (14)	−0.0042 (12)	0.0054 (12)	0.0042 (11)
C34	0.0499 (17)	0.0605 (19)	0.0481 (17)	−0.0026 (14)	0.0168 (14)	−0.0016 (14)

### Geometric parameters (Å, °)

**Table:**

Cu1—O1	1.9409 (16)	C11—C12	1.500 (5)
Cu1—O1W	1.9706 (18)	C12—H12A	0.9600
Cu1—O5	1.9278 (17)	C12—H12B	0.9600
Cu1—O9^i^	2.4505 (17)	C12—H12C	0.9600
Cu1—N5	2.032 (2)	C13—C14	1.509 (3)
O1—C1	1.274 (3)	C14—C15	1.382 (3)
O2—C1	1.244 (3)	C14—C19	1.386 (4)
O3—C9	1.230 (3)	C15—C16	1.379 (3)
O4—C11	1.216 (4)	C15—H15	0.9300
O5—C13	1.273 (3)	C16—C17	1.385 (4)
O6—C13	1.231 (3)	C16—H16	0.9300
O7—C21	1.220 (3)	C17—C18	1.381 (4)
O8—C23	1.203 (4)	C18—C19	1.378 (3)
O9—C30	1.234 (3)	C18—H18	0.9300
O1W—H11	0.841 (10)	C19—H19	0.9300
O1W—H12	0.836 (10)	C20—C21	1.496 (4)
N1—N2	1.308 (3)	C20—H20A	0.9600
N1—C5	1.401 (3)	C20—H20B	0.9600
N1—H1	0.876 (10)	C20—H20C	0.9600
N2—C10	1.319 (3)	C21—C22	1.462 (4)
N3—N4	1.296 (3)	C22—C23	1.480 (4)
N3—C17	1.406 (3)	C23—C24	1.499 (5)
N3—H3	0.875 (10)	C24—H24A	0.9600
N4—C22	1.323 (3)	C24—H24B	0.9600
N5—C29	1.334 (3)	C24—H24C	0.9600
N5—C25	1.336 (3)	C25—C26	1.376 (4)
N6—C30	1.333 (3)	C25—H25	0.9300
N6—C31	1.466 (3)	C26—C27	1.382 (4)
N6—C33	1.473 (3)	C26—H26	0.9300
C1—C2	1.499 (3)	C27—C28	1.390 (4)
C2—C3	1.384 (3)	C27—H27	0.9300
C2—C7	1.386 (3)	C28—C29	1.384 (3)
C3—C4	1.377 (3)	C28—C30	1.508 (3)
C3—H3A	0.9300	C29—H29	0.9300
C4—C5	1.389 (3)	C31—C32	1.527 (4)
C4—H4	0.9300	C31—H31A	0.9700
C5—C6	1.382 (4)	C31—H31B	0.9700
C6—C7	1.380 (3)	C32—H32A	0.9600
C6—H6	0.9300	C32—H32B	0.9600
C7—H7	0.9300	C32—H32C	0.9600
C8—C9	1.498 (4)	C33—C34	1.505 (4)
C8—H8A	0.9600	C33—H33A	0.9700
C8—H8B	0.9600	C33—H33B	0.9700
C8—H8C	0.9600	C34—H34A	0.9600
C9—C10	1.472 (4)	C34—H34B	0.9600
C10—C11	1.481 (4)	C34—H34C	0.9600
			
O5—Cu1—O1	176.95 (8)	C16—C15—H15	119.7
O5—Cu1—O1W	91.29 (8)	C14—C15—H15	119.7
O1—Cu1—O1W	88.01 (7)	C15—C16—C17	119.5 (2)
O5—Cu1—N5	90.85 (8)	C15—C16—H16	120.3
O1—Cu1—N5	90.32 (8)	C17—C16—H16	120.3
O1W—Cu1—N5	170.51 (8)	C18—C17—C16	120.5 (2)
O5—Cu1—O9^i^	86.30 (8)	C18—C17—N3	122.3 (2)
O1—Cu1—O9^i^	90.82 (7)	C16—C17—N3	117.2 (2)
O1W—Cu1—O9^i^	95.96 (7)	C19—C18—C17	119.4 (2)
N5—Cu1—O9^i^	93.40 (7)	C19—C18—H18	120.3
C1—O1—Cu1	110.97 (15)	C17—C18—H18	120.3
C13—O5—Cu1	119.32 (16)	C18—C19—C14	120.7 (2)
Cu1—O1W—H11	117 (3)	C18—C19—H19	119.6
Cu1—O1W—H12	118 (2)	C14—C19—H19	119.6
H11—O1W—H12	107 (3)	C21—C20—H20A	109.5
N2—N1—C5	120.5 (2)	C21—C20—H20B	109.5
N2—N1—H1	116 (2)	H20A—C20—H20B	109.5
C5—N1—H1	124 (2)	C21—C20—H20C	109.5
N1—N2—C10	121.0 (2)	H20A—C20—H20C	109.5
N4—N3—C17	121.0 (2)	H20B—C20—H20C	109.5
N4—N3—H3	116 (2)	O7—C21—C22	119.7 (2)
C17—N3—H3	123 (2)	O7—C21—C20	118.5 (3)
N3—N4—C22	121.1 (2)	C22—C21—C20	121.7 (3)
C29—N5—C25	118.3 (2)	N4—C22—C21	123.8 (2)
C29—N5—Cu1	119.66 (17)	N4—C22—C23	113.2 (2)
C25—N5—Cu1	122.06 (17)	C21—C22—C23	123.0 (2)
C30—N6—C31	124.6 (2)	O8—C23—C22	121.8 (3)
C30—N6—C33	118.6 (2)	O8—C23—C24	119.2 (3)
C31—N6—C33	116.1 (2)	C22—C23—C24	119.0 (3)
O2—C1—O1	124.2 (2)	C23—C24—H24A	109.5
O2—C1—C2	119.0 (2)	C23—C24—H24B	109.5
O1—C1—C2	116.8 (2)	H24A—C24—H24B	109.5
C3—C2—C7	119.0 (2)	C23—C24—H24C	109.5
C3—C2—C1	120.4 (2)	H24A—C24—H24C	109.5
C7—C2—C1	120.6 (2)	H24B—C24—H24C	109.5
C4—C3—C2	121.5 (2)	N5—C25—C26	122.5 (2)
C4—C3—H3A	119.2	N5—C25—H25	118.7
C2—C3—H3A	119.2	C26—C25—H25	118.7
C3—C4—C5	118.5 (2)	C25—C26—C27	119.0 (3)
C3—C4—H4	120.7	C25—C26—H26	120.5
C5—C4—H4	120.7	C27—C26—H26	120.5
C6—C5—C4	120.8 (2)	C26—C27—C28	119.2 (2)
C6—C5—N1	116.8 (2)	C26—C27—H27	120.4
C4—C5—N1	122.4 (2)	C28—C27—H27	120.4
C7—C6—C5	119.7 (2)	C29—C28—C27	117.7 (2)
C7—C6—H6	120.1	C29—C28—C30	121.7 (2)
C5—C6—H6	120.1	C27—C28—C30	120.1 (2)
C6—C7—C2	120.3 (2)	N5—C29—C28	123.3 (2)
C6—C7—H7	119.8	N5—C29—H29	118.4
C2—C7—H7	119.8	C28—C29—H29	118.4
C9—C8—H8A	109.5	O9—C30—N6	123.5 (2)
C9—C8—H8B	109.5	O9—C30—C28	117.2 (2)
H8A—C8—H8B	109.5	N6—C30—C28	119.2 (2)
C9—C8—H8C	109.5	N6—C31—C32	113.4 (2)
H8A—C8—H8C	109.5	N6—C31—H31A	108.9
H8B—C8—H8C	109.5	C32—C31—H31A	108.9
O3—C9—C10	119.2 (2)	N6—C31—H31B	108.9
O3—C9—C8	118.9 (3)	C32—C31—H31B	108.9
C10—C9—C8	121.9 (3)	H31A—C31—H31B	107.7
N2—C10—C9	123.8 (2)	C31—C32—H32A	109.5
N2—C10—C11	112.4 (3)	C31—C32—H32B	109.5
C9—C10—C11	123.8 (2)	H32A—C32—H32B	109.5
O4—C11—C10	120.8 (3)	C31—C32—H32C	109.5
O4—C11—C12	120.5 (3)	H32A—C32—H32C	109.5
C10—C11—C12	118.7 (3)	H32B—C32—H32C	109.5
C11—C12—H12A	109.5	N6—C33—C34	112.4 (2)
C11—C12—H12B	109.5	N6—C33—H33A	109.1
H12A—C12—H12B	109.5	C34—C33—H33A	109.1
C11—C12—H12C	109.5	N6—C33—H33B	109.1
H12A—C12—H12C	109.5	C34—C33—H33B	109.1
H12B—C12—H12C	109.5	H33A—C33—H33B	107.9
O6—C13—O5	125.2 (2)	C33—C34—H34A	109.5
O6—C13—C14	119.2 (2)	C33—C34—H34B	109.5
O5—C13—C14	115.6 (2)	H34A—C34—H34B	109.5
C15—C14—C19	119.2 (2)	C33—C34—H34C	109.5
C15—C14—C13	119.7 (2)	H34A—C34—H34C	109.5
C19—C14—C13	121.1 (2)	H34B—C34—H34C	109.5
C16—C15—C14	120.7 (2)		
			
O5—Cu1—O1—C1	−163.5 (14)	O6—C13—C14—C19	−177.3 (2)
O1W—Cu1—O1—C1	−86.75 (17)	O5—C13—C14—C19	3.0 (3)
N5—Cu1—O1—C1	83.91 (17)	C19—C14—C15—C16	−0.2 (4)
O1—Cu1—O5—C13	157.6 (14)	C13—C14—C15—C16	−179.9 (2)
O1W—Cu1—O5—C13	80.99 (19)	C14—C15—C16—C17	−0.4 (4)
N5—Cu1—O5—C13	−89.76 (19)	C15—C16—C17—C18	0.7 (4)
C5—N1—N2—C10	−179.9 (2)	C15—C16—C17—N3	179.8 (2)
C17—N3—N4—C22	−179.0 (2)	N4—N3—C17—C18	−7.1 (4)
O5—Cu1—N5—C29	−97.88 (18)	N4—N3—C17—C16	173.8 (2)
O1—Cu1—N5—C29	79.30 (18)	C16—C17—C18—C19	−0.4 (4)
O1W—Cu1—N5—C29	159.1 (4)	N3—C17—C18—C19	−179.5 (2)
O5—Cu1—N5—C25	83.2 (2)	C17—C18—C19—C14	−0.2 (4)
O1—Cu1—N5—C25	−99.6 (2)	C15—C14—C19—C18	0.4 (4)
O1W—Cu1—N5—C25	−19.8 (6)	C13—C14—C19—C18	−179.8 (2)
Cu1—O1—C1—O2	−0.9 (3)	N3—N4—C22—C21	1.0 (4)
Cu1—O1—C1—C2	179.77 (16)	N3—N4—C22—C23	−177.8 (2)
O2—C1—C2—C3	3.4 (4)	O7—C21—C22—N4	−1.2 (4)
O1—C1—C2—C3	−177.2 (2)	C20—C21—C22—N4	177.8 (3)
O2—C1—C2—C7	−177.1 (2)	O7—C21—C22—C23	177.6 (3)
O1—C1—C2—C7	2.3 (3)	C20—C21—C22—C23	−3.5 (4)
C7—C2—C3—C4	0.4 (4)	N4—C22—C23—O8	172.6 (3)
C1—C2—C3—C4	179.9 (2)	C21—C22—C23—O8	−6.2 (5)
C2—C3—C4—C5	−0.7 (4)	N4—C22—C23—C24	−7.6 (4)
C3—C4—C5—C6	0.9 (4)	C21—C22—C23—C24	173.5 (3)
C3—C4—C5—N1	−179.4 (2)	C29—N5—C25—C26	1.6 (4)
N2—N1—C5—C6	−178.1 (2)	Cu1—N5—C25—C26	−179.5 (2)
N2—N1—C5—C4	2.3 (4)	N5—C25—C26—C27	−1.2 (4)
C4—C5—C6—C7	−0.8 (4)	C25—C26—C27—C28	0.4 (4)
N1—C5—C6—C7	179.5 (2)	C26—C27—C28—C29	0.0 (4)
C5—C6—C7—C2	0.5 (4)	C26—C27—C28—C30	172.7 (2)
C3—C2—C7—C6	−0.3 (4)	C25—N5—C29—C28	−1.2 (4)
C1—C2—C7—C6	−179.8 (2)	Cu1—N5—C29—C28	179.79 (18)
N1—N2—C10—C9	1.6 (4)	C27—C28—C29—N5	0.5 (4)
N1—N2—C10—C11	−178.5 (2)	C30—C28—C29—N5	−172.1 (2)
O3—C9—C10—N2	−3.9 (4)	C31—N6—C30—O9	171.9 (2)
C8—C9—C10—N2	175.9 (3)	C33—N6—C30—O9	1.7 (4)
O3—C9—C10—C11	176.3 (3)	C31—N6—C30—C28	−11.3 (4)
C8—C9—C10—C11	−4.0 (4)	C33—N6—C30—C28	178.6 (2)
N2—C10—C11—O4	174.5 (3)	C29—C28—C30—O9	118.7 (3)
C9—C10—C11—O4	−5.6 (5)	C27—C28—C30—O9	−53.7 (3)
N2—C10—C11—C12	−3.5 (4)	C29—C28—C30—N6	−58.3 (3)
C9—C10—C11—C12	176.4 (3)	C27—C28—C30—N6	129.2 (3)
Cu1—O5—C13—O6	−0.8 (4)	C30—N6—C31—C32	110.6 (3)
Cu1—O5—C13—C14	178.80 (15)	C33—N6—C31—C32	−79.0 (3)
O6—C13—C14—C15	2.4 (4)	C30—N6—C33—C34	91.6 (3)
O5—C13—C14—C15	−177.3 (2)	C31—N6—C33—C34	−79.4 (3)

Symmetry codes: (i) −*x*+1, *y*−1/2, −*z*+3/2.

### Hydrogen-bond geometry (Å, °)

**Table:**

*D*—H···*A*	*D*—H	H···*A*	*D*···*A*	*D*—H···*A*
O1w—H11···O2^ii^	0.84 (1)	1.88 (1)	2.700 (2)	163 (4)
O1w—H12···O6^ii^	0.84 (1)	1.88 (1)	2.705 (3)	171 (3)
N1—H1···O3	0.88 (1)	1.84 (2)	2.552 (3)	138 (3)
N3—H3···O7	0.88 (1)	1.85 (2)	2.559 (3)	137 (3)

Symmetry codes: (ii) −*x*+1, −*y*+1, −*z*+1.
